# Enhanced Chromatin Accessibility and Recruitment of JUNB Mediate the Sustained IL-4 Expression in NFAT1 Deficient T Helper 2 Cells

**DOI:** 10.1371/journal.pone.0022042

**Published:** 2011-07-25

**Authors:** Jun-Seock Son, Chang-Suk Chae, Ji-Sun Hwang, Zee Yong Park, Sin-Hyeog Im

**Affiliations:** School of Life Sciences and Immune Synapse Research Center, Gwangju Institute of Science and Technology, Gwangju, Republic of Korea; Institute of Genetics and Molecular and Cellular Biology, France

## Abstract

Nuclear factor of activated T cells (NFAT) is a family of transcription factors composed of five proteins. Among them, NFAT1 is a predominant NFAT protein in CD4^+^ T cells. NFAT1 positively regulates transcription of a large number of inducible cytokine genes including IL-2, IL-4, IL-5 and other cytokines. However, disruption of NFAT1 results in an unexpected increase of IL-4. In this study, we have investigated the role of NFAT1 in regulation of *IL-4* gene expression in T helper 2 cells (Th2) from an epigenetic viewpoint. NFAT1 deficient Th2 cells showed a sustained IL-4 expression while wild type (WT) cells reduced its expression. We tested whether epigenetic maintenance and changes in the chromatin architecture of IL-4 promoter locus play a role in differential IL-4 transcription between in WT and NFAT1 deficient Th2 cells. Compared with WT, NFAT1 deficient CD4^+^ Th2 cells exhibited enhanced chromatin accessibility with permissive histone modification and DNA demethylation in the IL-4 promoter region. Transcription factors bound to IL-4 promoter region in the absence of NFAT1 were identified by Micro-LC/LC-MS/MS analysis. Among the candidates, preferential recruitment of JUNB to the IL-4 promoter was confirmed by chromatin immunoprecipitation analysis. Overexpression of JUNB together with SATB1 synergistically upregulated IL-4 promoter activity, while knockdown JUNB significantly reduced IL-4 expression. Our results suggest that the prolonged IL-4 expression in NFAT1 deficient Th2 cells is mediated by preferential binding of JUNB/SATB1 to the IL-4 promoter with permissive chromatin architecture.

## Introduction

T cell receptor (TCR) signaling drives T lymphocyte gene expression and activation of nuclear factor of activated T cells (NFAT) [1]. NFAT proteins regulate transcription of a large number of inducible genes in immune system including diverse cytokines, costimulatory factors and their receptors [1], [2]. NFATs are rapidly activated by calcineurin phosphatase after TCR engagement, and return back to inactive phosphorylated states by several kinases within a short period of time [3], [4], [5], [6]. The dephosphorylation and phosphorylation of NFAT regulates its functional activity by affecting its subcellular localization, duration of nuclear residence, interacting partners, DNA binding activity and its transcriptional activity [4]. The NFAT family of transcription factors is composed of five proteins. Among them, NFAT1 is a predominant NFAT protein in T cells and accounts for 90% of total NFAT DNA binding activity in wild type T cells [7]. NFATs mainly work as transcriptional activators during the immediate early time and determine the subsequent gene expression program. NFAT1 positively regulates transcription of a large number of inducible cytokine genes including IL-2, IL-4, IL-5 and other cytokines as well [1]. However, disruption of NFAT1 results in an unexpected increase of IL-4 upon stimulation [8]. Mice lacking NFAT1 show a modest splenomegaly, hyperproliferation of T and B cells and dysregulated production of IL-4 [8], [9], [10], [11], [12], [13]. Furthermore, in the absence of NFAT1 and NFAT4, naïve CD4^+^ T cells intrinsically differentiate into the T helper type 2 (Th2) cell direction, even in the absence of endogenous IL-4, and are hyperresponsive to TCR-mediated activation [14]. Although some phenotypes of NFAT1/4-deficient mice are due to defect in lymphocyte apoptosis [15], the cause of the various abnormalities still remains unknown.

Optimal gene expression requires recruitment of specific transcription factors to the regulatory regions (enhancers and promoters) of their target gene loci with permissive chromatin structure. Changes in the remodeling of chromatin structure are accompanied by epigenetic modifications such as changes in post-translational modification of specific histone residues and DNA methylation statues of CpG island. Epigenetic modifications affect gene transcription by altering the accessibility of distinct DNA regions to transcription factors and other DNA binding molecules [16], [17], [18]. Among histone modifications, acetyl histone H3 lysine 9/14 (AcH3K9/14) and dimethyl histone H3 lysine 4 (H3K4me2) are associated with actively transcribed genes required for T cell function and development, whereas trimethyl histone H3 lysine 27 (H3K27me3) is associated with silent genes [19]. In addition, methylated CpG inhibits the access of transcriptional machinery and decreases transcriptional activity [20], [21], [22]. Several groups have observed that epigenetic regulation is crucial for controlling *IL-4* gene expression [23], [24], [25], [26], [27].

Special AT-rich sequence-binding protein-1 (SATB1) is a global chromatin organizer and transcription factor. It plays a key role in forming a transcriptionally poised chromatin [28], [29] to induce gene expression in response to physiological stimuli [30]. SATB1 also organizes the IL-4 locus into distinct chromatin loops by tethering matrix associated regions (MARs) to the nuclear matrix at fixed distances. Upon Th2 cell activation, SATB1 is also required for the expression of IL-4, IL-5, and IL-13 through formation of transcriptionally active chromatin structure [29].

In this study, we have investigated the underlying mechanism of *IL-4* gene expression in the absence of the key transcription factor NFAT1 in Th2 cells. We and others have found that Th2 cells lacking NFAT1 still express high levels of IL-4. Prolonged expression of IL-4 was observed in NFAT1 deficient Th2 cells upon TCR engagement at late time points compared with wild type Th2 cells. We tested the possibility that NFAT1 deficiency may bring a change in the chromatin architecture in the IL-4 promoter and recruitment of other transcription factors may replace the transactivity of NFAT1 to mediate prolonged IL-4 expression. Here we show that the sustained IL-4 expression is mediated by a permissive chromatin change and by the recruitment of JUNB/SATB1/coactivator complex upon TCR stimulation in NFAT1-deficient Th2 cells.

## Results

### IL-4 expression is sustained in NFAT1 deficient Th2 cells compared with WT

**Figure 1:**
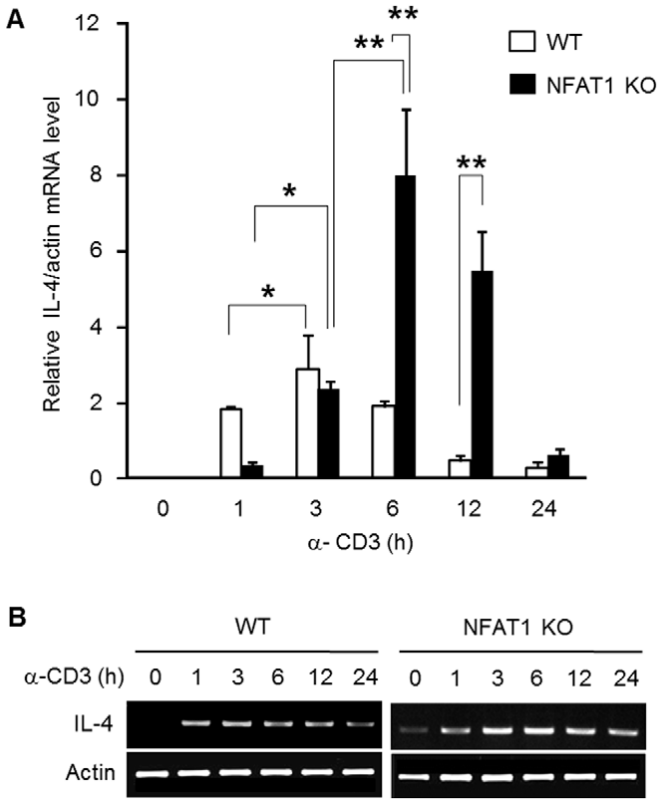
Sustained IL-4 expression in NFAT1 deficient Th2 cells. Th2 cells differentiated *in vitro* from WT or NFAT1 KO mice were stimulated with anti-CD3 (α-CD3) for indicated time periods and IL-4 mRNA levels were measured by quantitative RT-PCR by normalizing with β-actin levels (A). PCR products were visualized on ethidium bromide-stained agarose gels (B). Data shown are the mean ± SEM from four separate experiments and * P<0.05, ** P<0.01.

NFAT1 positively regulates *IL-4* gene transcription in CD4^+^ T cells. However, disruption of NFAT1 results in an unexpected prolonged increase of IL-4 upon stimulation. Anti-CD3 (α-CD3) stimulation significantly increased IL-4 transcripts in NFAT1 deficient CD4^+^ T cells than in WT mice [8], [14]. To elucidate the underlying mechanism of IL-4 expression in the absence of NFAT1, Th2 cells differentiated from wild type (WT) or lacking NFAT1 (NFAT1 KO) mice were stimulated with anti-CD3 for the indicated time periods and the IL-4 at mRNA and protein levels were determined by quantitative real-time PCR and ELISA, respectively. In line with previously published data [8], NFAT1 deficient Th2 cells maintained much higher levels of IL-4 expression than WT cells till later time points (Fig. 1). The IL-4 mRNA level showed a peak expression at 3 h after stimulation in WT cells followed by a rapid decline. Since NFAT1 is rapidly activated through dephosphorylation within 1 h and then returned to the phosphorylated inactive form (Fig. S1A), *IL-4* gene transcription at the early time (<1 h) might be mainly mediated by NFAT1 activation. In NFAT1 deficient T cells, however, the IL-4 mRNA levels were progressively increased until 6 h and were then maintained at much higher level compared with WT (Fig. 1). Enhanced IL-4 protein level was also observed in Th2 cells lacking NFAT1 compared with WT Th2 cells analyzed by ELISA (Fig. S1B).

### The IL-4 promoter of NFAT1 deficient Th2 cells has more permissive chromatin structure

**Figure 2:**
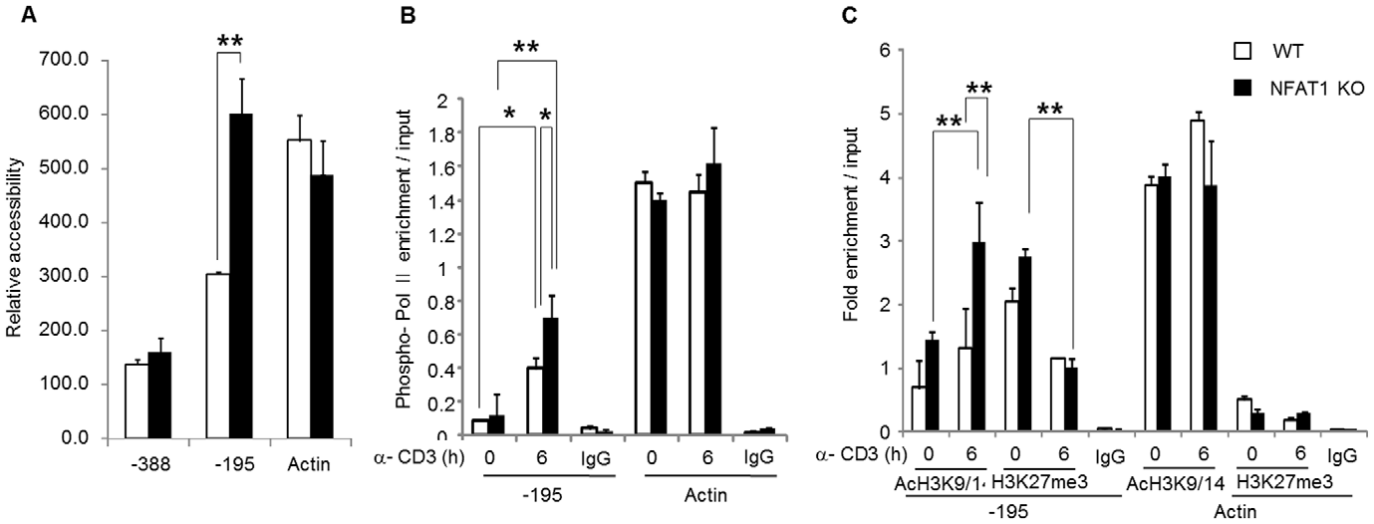
NFAT1 deficient Th2 cells have more permissive chromatin structure at the IL-4 promoter. (A) For chromatin accessibility analysis, Th2 cells differentiated *in vitro* from WT or NFAT1 KO mice were stimulated with anti-CD3 for 6 h. Nuclei isolated from each group and were incubated with or without 50 U Mnase. Fifty nanograms of genomic DNA from each treatment were subjected to real-time PCR analysis with indicated primer sets covering IL-4 promoter. The Ct values generated were converted to DNA concentrations by using the standard curve. MNase accessibility was expressed as a relative value of undigested genomic DNA and plotted for each primer set. Graphs depict the PCR products from digested samples normalized to the PCR products from undigested samples and show mean ± SEM, n = 3 and ** P<0.01 for four separate experiments. To measure the mounts of recruited Pol II and modified histone molecules at the IL-4 promoter, ChIP analysis was performed with antibodies specific for RNA Pol II (B) and acetyl histone H3 lysine 9/14 (AcH3K9/14), trimethyl histoneH3 lysine 27 (H3K27me3) or isotype matched control IgG (C). Relative amount of enriched DNA by ChIP was measured by quantitative RT-PCR using the primers spanning the indicated IL-4 promoter region or β-actin region. The graph represents mean ± SEM, from four separate experiments and * P<0.05, ** P<0.01.

IL-4 expression is mainly mediated by the NFAT1 at immediately early time points (Fig. 1A). However, underlying mechanism for higher IL-4 expression in NFAT1 deficient CD4^+^ T cells is still unknown. To test the role of epigenetic change in these states, we compared chromatin accessibility to micrococcal nuclease (MNase) at the IL-4 promoter between WT and NFAT1 deficient Th2 cells. Nuclei isolated from Th2 cells of WT and NFAT1 KO mice were stimulated for 6 h with anti-CD3 and were incubated with MNase for 5 min at room temperature. The amount of amplified genomic DNA from each treatment was quantitatively measured by real-time PCR with primer sets specific for IL-4 promoter (Table 1). IL-4 promoter was more accessible in NFAT1 deficient Th2 cells than in WT (Fig. 2A). To address transcriptional activity as a marker for active chromatin, the amount of recruited RNA polymerase II (Pol II) to the IL-4 promoter and within the body of the *IL-4* gene (Exon1) was assessed by ChIP assay using phospho-Pol II antibody. The enrichment of the phospo-Pol II molecule was observed at promoter (Fig. 2B) and within the body of the *IL-4* gene (data not shown) in the NFAT1 deficient Th2 cells compared with the WT cells. To further confirm whether the differential chromatin accessibility at the IL-4 promoter between WT and NFAT1 KO Th2 cells is accompanied by epigenetic modification, we performed ChIP analysis with specific antibodies for modified histone molecules by quantitative real-time PCR analysis. In general, modification of acetylated H3 at lysine residue 9 and 14 (AcH3K9/14) and trimethylated H3 at lysine 27 (H3K27me3) are well correlated with actively transcribed or silenced region, respectively. In both WT and NFAT1 deficient Th2 cells, amounts of AcH4K9/14 were significantly increased upon stimulation, whereas H3K27me3 levels were decreased at the IL-4 promoter (Fig. 2C). However, NFAT1 deficient Th2 cells showed significantly elevated AcH3K9/14 binding levels compared with WT cells at the IL-4 promoter (Fig. 2C). As a control we also analyzed the actin promoter region. Regardless of stimulation, the amount of recruited levels of AcH4K9/14 or H3K27me3 at the action promoter was similar between WT and NFAT1 deficient Th2 cells (Fig. 2C). These results suggest that more permissive chromatin structure in NFAT1 deficient CD4^+^ Th2 cells compared with WT cells at the IL-4 promoter is related with sustained higher IL-4 expression.

**Table 1:** List of oligonucleotide primer sequence (5′ to 3′).

	Sense	Antisense	Product size (bp)
CHART (Chromatin accessibility real time PCR)			
-393	TCAAGGCAGACTTTCTTGATATTACTCTGT	AATCAGCACCTCTCTTCCAGGAGAA	279
-195	GTGTTTCATTTTCCAATTGGTCT	AATCAGCACCTCTCTTCCAGGAGAA	81
actin	AGCACAGCTTCTTTGCAGCTCCTT	AGGACCCTGCAGTGAGGTACTA	117
ChIP (Chromatin immunoprecipitation)			
-278	GATAAGATTAGTCTGAAAGGCCGATTATG	AATCAGCACCTCTCTTCCAGGAGAA	164
-195	GTGTTTCATTTTCCAATTGGTCT	AATCAGCACCTCTCTTCCAGGAGAA	81
actin	AGCACAGCTTCTTTGCAGCTCCTT	AGGACCCTGCAGTGAGGTACTA	117
EMSA			
ikB	AGCTTCAGAGGGGACTTTCCGAGAGG	CCTCTCGGAAAGTCCCCTCTGAAGCT	
P1	GGAGGGGTGTTTCATTTTCCAATTGGTCTGATTTC	GAAATCAGACCAATTGGAAAATGAAACACCCCTCC	
P2	CTGGTGTAATAAAATTTTCCAATGTAAACTCATTT	AAATGAGTTTACATTGGAAAATTTTATTACACCAG	
RT-PCR			
IL-4	CAACGAAGAACACCACAGAG	GGACTTGGACTCATTCATGG	192
IL-2	ACCTCTGCGGCATGTTCTGGATTTGACTC	CCTCAGAAAGTCCACCACAGTTGCT	169
JunB	AGGTGAAGACACTCAAGGCTGAGAA	TGACATGGGTCATGACCTTCTGCTT	102
Actin	CTGTGGCATCCATGAAACTACATTCAAT	AGGAGGAGCAATGATCTTGATCTTCA	145
aLuc constructs			
	GAAGATCTAAATCTTCAACCTAGCCCAG	GGGAAGCTTTTGAGCTTTGTCCCTAGTCC	977
Pyrosequencing analysis			
	GATATCAGAGTTTCCAAG	AGAGAAGGAAGAGGTCACAGGT	140
MeDIP			
IL-4	ACAAACTTGTAAGATCAGCTGGTCT	ATCTACAAAGTTTCAGCATAGGAAATTA	322
FoxP3	TGCAAAGACCCTAGCTTTACACTTCAGT	ACCAAACCATGGACCCTGAGAAAAT	204
actin	GGCCAGCGTTTGCCTTTTATGGTAATAAT	TATAGCCTTCTTTTGTGTCTTGATAGTT**CG**	181

a Luc, luciferase.

### CpG sites at the IL-4 promoter of NFAT1 deficient Th2 cells are preferentially demethylated

**Figure 3:**
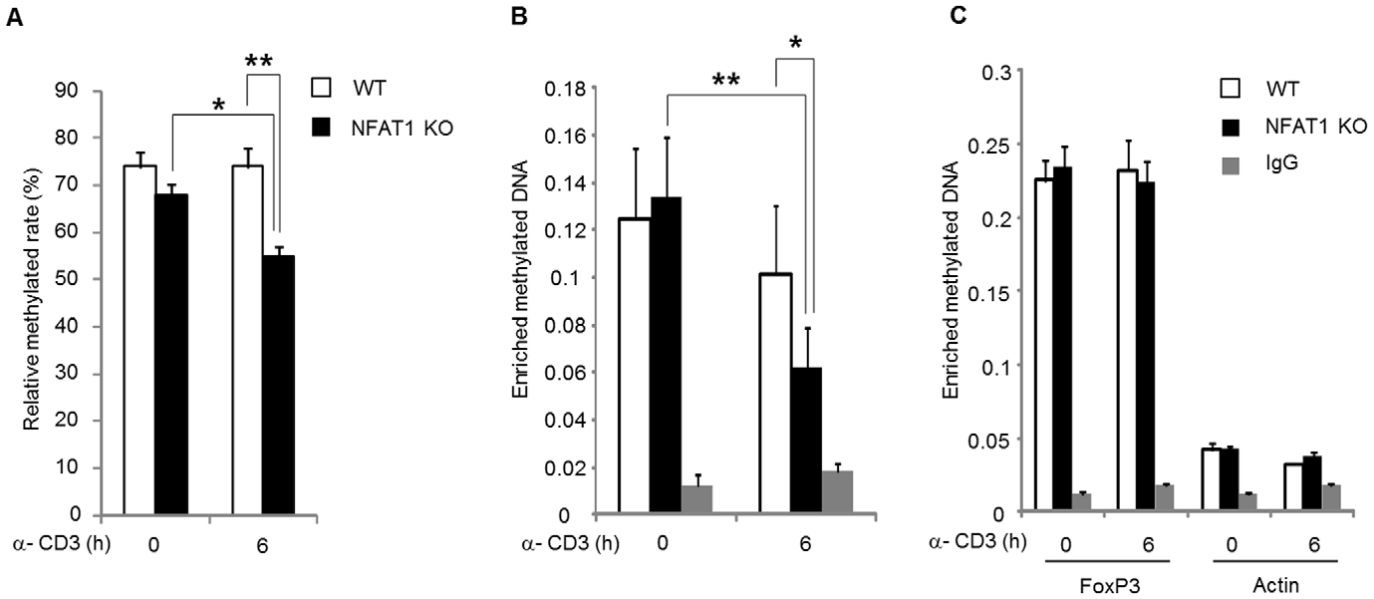
Increased DNA demethylation status at the IL-4 promoter of NFAT1 deficient Th2 cells. Th2 cells from WT or NFAT1 KO mice were stimulated with anti-CD3 for 6 h or left without stimulation. DNA methylation state at the IL-4 promoter was analyzed by pyrosequencing (Fig. S2) and ChIP analysis using anti-5mC antibodies specific for methylated DNA at IL-4 promoter (B). FoxP3 and β-actin locus were tested as positive or negative control, respectively for methylated DNA experiment, (C). The relative amount of enriched methylated DNA was assessed by real-time PCR analysis. 10% of the input sample was used for PCR amplification and normalized to the input samples from three independent experiments and * P<0.05, ** P<0.01.

Generally hypermethylation of DNA at the gene promoters or CpG island is associated with transcriptional repression while hypomethylation is associated with actively transcribed genomic locus. To test whether the differential methylation statute of IL-4 promoter between WT and NFAT1 deficient Th2 cells is involved in differential IL-4 expression between them, we analyzed the DNA methylation status at the IL-4 promoter by pyrosequencing. Under unstimulated condition, little difference in the methylation status was observed between NFAT1 deficient (68%) and WT (74%) Th2 cells. Six hours after stimulation, however, the level of methylated DNA in the IL-4 promoter of NFAT1 deficient Th2 cells was significantly decreased (55%) than that of WT (74%) Th2 cells (Fig. 3A and Fig. S2). To confirm this observation, we next assessed the levels of enriched methylated DNA at the IL-4 locus by methylated DNA immunoprecipitation assay. In line with the pyrosequencing data, a significant decrease of demethylated DNA at the IL-4 promoter was observed in NFAT1 deficient cells compared with WT Th2 cells (Fig. 3B). As a control, methylated DNA levels were also measured at the FoxP3 and beta-actin gene locus as controls for repressed or active gene, respectively. These results suggest that DNA hypomethylation at the IL-4 promoter of NFAT1 deficient Th2 cells is related with sustained higher IL-4 expression.

### Preferential recruitment of JUNB to the IL-4 promoter in NFAT1 deficient Th2 cells

**Figure 4:**
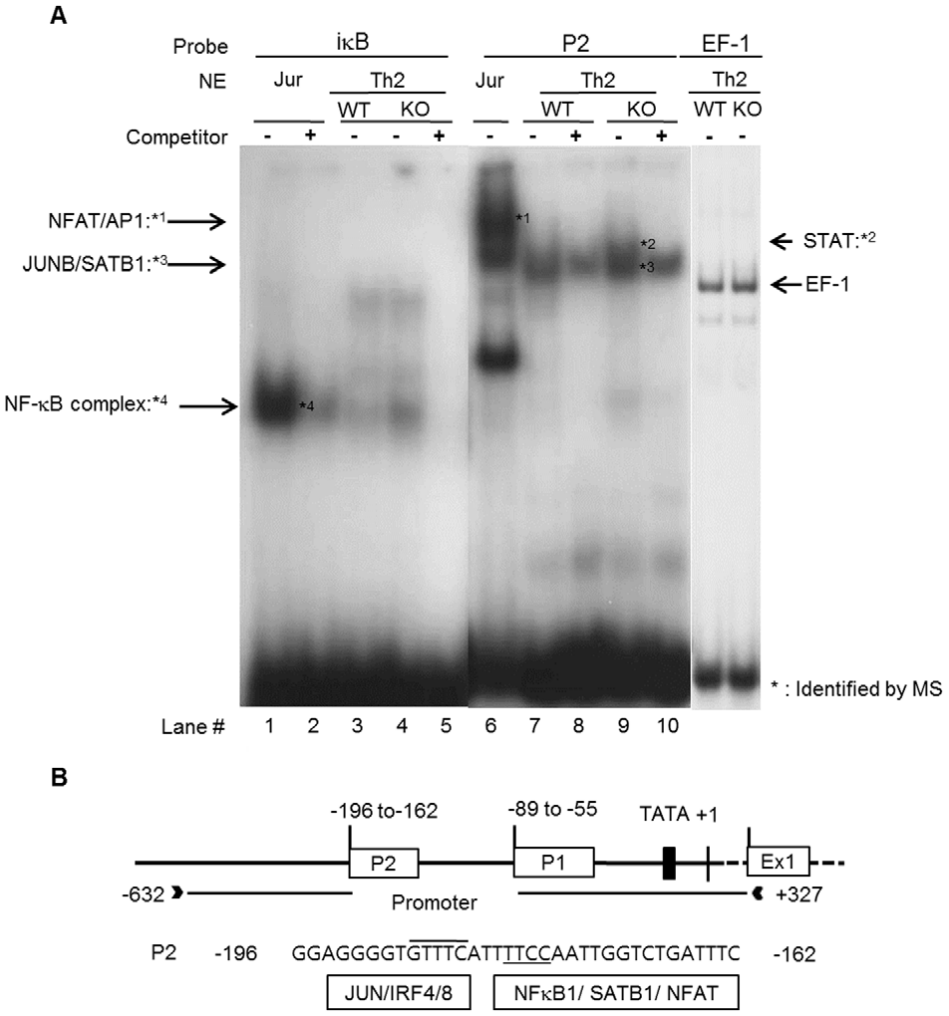
Identification and analysis of IL-4 promoter-binding transcription factors. EMSA assay was carried out with nuclear extracts prepared from Th2 cells of WT and NFAT1 KO or from Jurkat cells that were stimulated with anti-CD3 for 6 h or PMA/I for 2 h, respectively (A). ^32^P-labelled P2 probes were incubated with indicated nuclear extracts. IκB probe containing κB element was used as a positive control and 100 pmol unlabeled iκB probe was used as a competitor to interfere the complex formation. (−) or (+) indicates in the absence or presence competitor, respectively. The DNA binding complexes that appeared or were enhanced after stimulation are indicated. Arrows indicate the identified proteins that bound to the corresponding gel band. Equal loading of nuclear extracts was controlled by using the binding site for the constitutive factor EF-1 as a probe. Identification of DNA-binding transcription factors was carried out by micro-LC/LC-MS/MS analysis as described in Material and Methods. (B) Predicted DNA binding elements for the P2 region of IL-4 promoter.

The higher expression level of IL-4 in NFAT1 deficient Th2 cells than WT cells suggests that transcription factors other than NFAT1 may bind to the accessible IL-4 promoter to transactivate IL-4 expression. To identify the transcription factors bound at IL-4 promoter we performed EMSA and Micro-LC/LC-MS/MS analysis. IL-4 promoter contains binding sites for important transcription factors [31], [32], [33] (Fig. 4B). Jurkat T cells (human T cell lymphoma cell line) or Th2 cells from WT or NFAT1 deficient cells were stimulated by PMA/ionomycin or anti-CD3 for 6 h, respectively and then nuclear extracts were prepared. Oligomers corresponding to P2 (-196 to -163) locus of IL-4 promoter were incubated with the isolated nuclear proteins and then EMSA analysis was performed. IκB probe was used as a positive control for the binding of the NF-κB complex and EF-1 probe was used as a loading control. The regulatory element P2 was shown to interact with nuclear proteins of Th2 cells (Fig. 4A). Bands showing different mobility pattern between the WT and NFAT1 deficient cells were cut and then the identity of binding proteins were analyzed by Micro-LC/LC-MS/MS analysis (Table 2). Jurkat nuclear extract showed the NF-κB complex (lane 1) and NFAT/AP1 complex (Lane 6) (Fig. 4A). Nuclear extracts of NFAT1 deficient Th2 cells showed a much stronger signal of STAT (lane 9), SATB1/JUNB (lane 9) complex incubated with P2 element than WT Th2 cells (Fig. 4A lane 12). Formation of EF-1 binding complex was same between WT and NFAT1. This result suggests that preferential binding of JUNB/SATB1 to the P2 regulatory element in NFAT1 deficient Th2 cells may mediate sustained IL-4 expression.

**Table 2:** Proteins of interest and their peptide sequences detected by micro LC/LC-MS/MS analysis.

Protein	Peptide sequence
Special AT-rich sequence-binding protein-1 (SATB1)	R.LLAQQSLNQQYLNHPPPVSR.S R.AGISQAVFAR.V R.TQGLLSEILR.K
Nuclear factor kappa-B (NF-κB)	R.YVCEGPSHGGLPGASSEK R.RLEPVVSDAIYDSK.A K.VIVQLVTNGK.N
Jun-B oncogene (JUNB)	R.GPGPEGSGAGSYFSGQGSDTGASLK.L R.GASAFKEEPQTVPEAR.S K.AENAGLSSAAGLLR.E
Nuclear factor of activated T-cells (NFAT1)	R.IEVQPKPHHR.A
Jun oncogene (c-Jun)	R.ELTDTLQAETDQLEDEKSALQTEIANLLK.E
Signal-transducer and activator of transcription protein (STATs)	R.GQATQLLEGLVQELQK.K R.LITQDTENELKK.L R.EAQTLQQYR.V K.FLEQVHQLYDDSFPMEIR.Q R.FHDLLSQLDDQYSR.F R.FHDLLSQLDDQYSR.F R.FSLENNFLLQHNIR.K R.FNQAQEGNIQNTVMLDK.Q R.ELLNSIELTQNTLINDELVEWK.R K.FTYEPDPITK.N R.TFLLFQQLIQSSFVVER.Q K.LLGPNAGPDGLIPWTR.F R.EGAITFTWVER.S K.TELISVSEVHPSR.L

- Number indicates the frequency of unique peptide of the detected protein.

**Figure 5:**
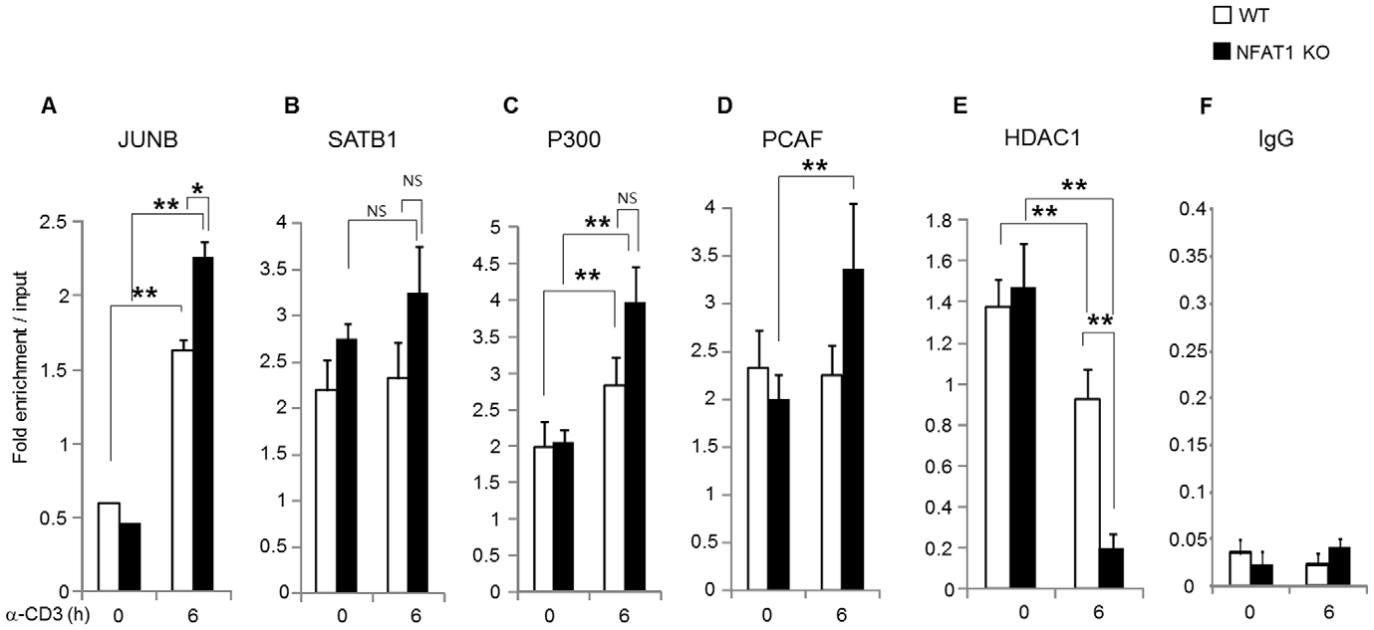
*In vivo* binding of JUNB, SATB1 and cofactors to the IL-4 promoter. Th2 cells from WT or NFAT1 KO mice were stimulated with anti-CD3 for 6 h or left without stimulation. The relative amount of DNA-protein complex enriched at the P2 locus of IL-4 promoter was analyzed by ChIP using control normal IgG (F) or specific antibodies for JUNB (A), SATB1 (B), and other cofactors such as P300 (C), PCAF (D) and HDAC1 (E). Relative enrichment at the P2 locus of IL-4 promoter in the precipitated samples compared to total chromatin (input) is shown. Data are representative of three independent experiments and * P<0.05, ** P<0.01.

To further test whether the identified proteins by Micro-LC/LC-MS/MS analysis are also recruited to the IL-4 promoter (P2 region) *in vivo*, ChIP assay was performed. WT and NFAT1 deficient Th2 cells were stimulated with anti-CD3 for 6 h and DNA-protein complex were enriched by use of specific antibodies for SATB1, JUNB, and other cofactors such as P300, PCAF, and HDAC1. The relative amounts of enriched P2 region from each ChIP experiment were measured by quantitative RT-PCR. Indeed, *in vivo* binding of JUNB, P300, and PCAF to the P2 region of IL-4 promoter were enriched in a stimulation-dependent manner (Fig. 5). Compared with WT cells, significant increase of JUNB (Fig. 5A) and PCAF (Fig. 5D) binding in NFAT1 deficient cells was observed while the level of SATB1 binding (Fig. 5B) was similar between WT and NFAT1 deficient cells regardless of stimulation. We also analyzed the relative levels of recruited HDAC1 as a SATB1-interacting partner of repressor complex. Interestingly, a significant decrease of HDAC1 binding to the P2 region of IL-4 promoter was observed in NFAT1 deficient cells compared with WT cells in a stimulation-dependent manner (Fig. 5E). As a negative control for ChIP experiments, isotype matched normal IgG data was included (Fig. 5F). This result suggests that *in vivo* binding of JUNB/SATB1 together with transcriptional coactivators (P300 and PCAF) complex to the IL-4 promoter mediates IL-4 expression in NFAT1 deficient Th2 cells.

### SATB1 and JUNB synergistically transactivate the IL-4 promoter

**Figure 6:**
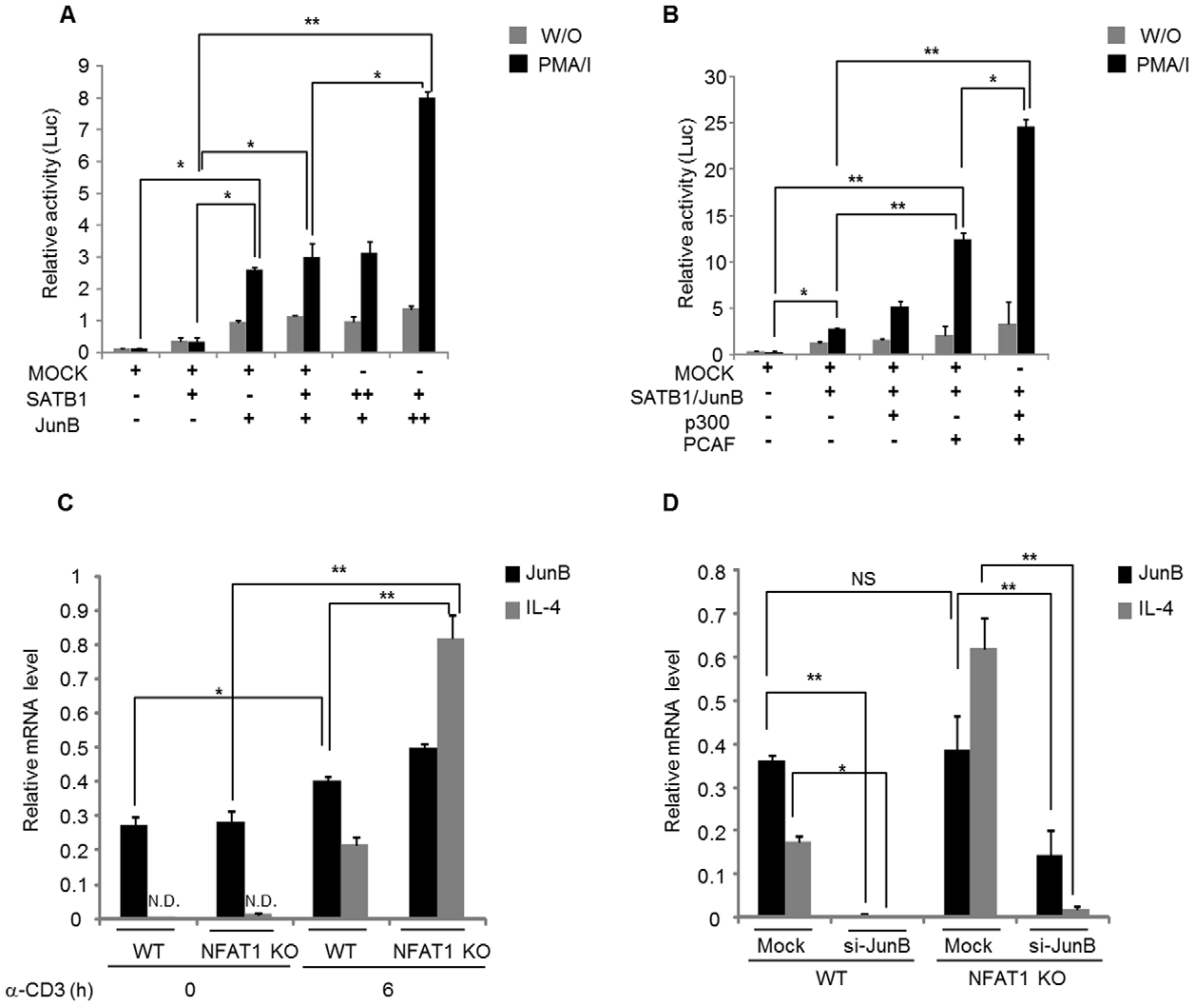
SATB1 and JUNB synergistically transactivate IL-4 promoter with other cofactors. (A) IL-4 reporter construct was transfected into HEK cells in the presence of different amount (0, 0.2 µg (+) or 0.4 (++) µg) of SATB1 or JunB expression plasmid alone or both of them, and then luciferase activity was measured. In each transfection, 0.1μg of TK-luciferase plasmid was added as an internal control for normalization of transfection efficiency. Transfected cells were harvested after 24 h in reporter lysis buffer, and analyzed for luciferase activity. (B) IL-4 reporter construct was transfected into HEK cells, in duplicate, with 0.2 µg of STATB1/JunB expression plasmids, in the absence or presence of 0.2 µg of p300 or/and PCAF expression plasmids. In each transfection, 0.1 µg of TK-luciferase plasmid was added and total DNA was maintained at 0.8 µg by addition of the appropriate amounts of pcDNA3 control plasmid. After 24 h, cells were harvested and analyzed for firefly and Renilla luciferase activities. Values were normalized to Renilla activities. The graphs in A-B represents mean ± SEM, n = 3 and * P<0.05, ** P<0.01. Data are representative of three independent experiments. (C) Th2 cells from WT or NFAT1 deficient were left without stimulation or stimulated for 6 h. The expression levels of JunB and IL-4 were measured by RT-PCR. (D) Th2 cells from WT and NFAT1 deficient mice were transfected with scrambled mock siRNA (Mock) or JunB specific siRNA (si-JunB) and then specific knockdown efficiency of JunB and its effect on IL-4 expression level was measured by RT-PCR. Data are representative of three independent experiments and * P<0.05, ** P<0.01.

To test the functional role of SATB1 and JUNB recruitment *in vivo* to the IL-4 promoter, we performed IL-4 reporter analysis by measuring luciferase activity driven by IL-4 promoter. IL-4 reporter construct was transfected into HEK cells in the presence of SATB1 or JunB expression plasmids alone or together, and then luciferase activity was measured. SATB1 alone failed to activate IL-4 promoter activity while JunB alone significantly activated it (Fig. 6A). However, co-transfection of SATB1 with JunB synergistically transactivated IL-4 promoter activity in a dose dependent manner (Fig. 6A). Since transcription coactivators such as P300 and PCAF also bind to the IL-4 promoter *in vivo* (Fig. 5), we tested whether expression of transcription coactivators (P300 and PCAF) together SATB1/JunB could further enhance the IL-4 promoter activity. Indeed, overexpression of the PCAF and/or p300 cofactors significantly enhanced transactivation activity (Fig. 6B). In the formation of transcriptional activation complex, JUNB may play a pivotal role through interaction with SATB1 and coactivators since SATB1 alone failed to activate IL-4 promoter activity while overexpression of JunB enhanced it in a dose-dependent manner (Fig. 6A). To further validate the JUNB-dependent *IL-4* gene activation we tested the knockdown effect of JUNB by using JunB siRNA (si-JunB) in WT and NFAT1 deficient Th2 cells. We first confirmed that stimulation of Th2 cells with anti-CD3 significantly upregulated IL-4 expression (Fig. 6C and Fig. S4A). Then cells were transfected with scrambled or si-JunB. Overexpression of si-JunB successfully reduced JunB levels (Fig. 6D and Fig. S4B), which significantly reduced IL-4 transcript levels compared with mock siRNA treatment (Fig. 6D). These results suggest that JUNB plays pivotal role to induce IL-4 expression through coordinated interaction with other coactivators in Th2 cells.

## Discussion

NFAT1 is a crucial transcription transcriptional activator and regulates expression of cytokines and other inducible genes in immune cells. However, NFAT1 deficiency results in hyperresponsiveness and sustained expression of IL-4 in CD4^+^ Th2 cells upon TCR stimulation. In this study, we have investigated the molecular mechanism of sustained *IL-4* gene expression in NFAT1 deficient T helper 2 cells. Our results showed that sustained IL-4 expression in NFAT1 deficient Th2 cells is mediated by enhanced chromatin accessibility with permissive histone modification and DNA demethylation in the IL-4 promoter. Furthermore, preferential recruitment of JUNB/SATB1 with transcriptional coactivators (PACF and P300) to the IL-4 promoter mediates continual IL-4 expression in the absence of NFAT1.

NFAT is a family of transcription factors composed of five proteins. These include NFAT1 (NFATp, NFATc2), NFAT2 (NFATc, NFATc1), NFAT3 (NFATc4), NFAT4 (NFATx, NFATc3), and NFAT5 (TonEBP: tonicity element binding protein or OREBP: osmotic response element binding protein). The role of NFAT1 as a transcriptional activator has been well documented in regulation of diverse target genes including cytokines and costimulatory factors [1]. Depending on their binding partners, however, the transcriptional activity of NFATs can be varied as an activator or inhibitor. For example, coupling with AP-1 (composed of Fos and Jun proteins) or MEF2 activates gene expression while in the absence of cooperative binding with AP-1 in the nucleus or alternative binding with histone deacetylases (HDACs) turns on a negative regulatory gene program [34], [35]. Specific role of NFAT proteins were investigated by knockout mice models [36]. NFAT1 is a predominant NFAT protein in T cells [7] and mainly enhances cytokine expression including IL-4 by lowering the threshold for TCR-mediated activation [14]. Interestingly, however, disruption of NFAT1 enhanced IL-4 expression especially at the later stage of stimulation. What is the underlying mechanism of *IL-4* gene regulation in the absence of NFAT1? In addition, how come *IL-4* gene expression is more sustained in NFAT1 deficient Th2 cells than WT? Since NFAT1 also works as a transcription repressor by interacting with repressor proteins we hypothesized that in the absence of NFAT1, changes in the chromatin structure at the IL-4 promoter might be different between WT and NFAT1 deficient Th2 cells. Indeed, chromatin accessibility at the IL-4 promoter was significantly increased in NFAT1 deficient cells compared with WT cells (Fig. 2A), which is further confirmed by the enhanced recruitment of phospho-Pol II in NFAT1 deficient Th2 cells (Fig. 2B). In addition, permissive chromatin architecture related with epigenetic modification showed a close correlation. Compared with WT cells, there was enhanced modification of active chromatin marker histones (AcH3K4Me2, AcH3K9/14). In addition, reduction of methylated DNA and H3K27me3 was observed in NFAT1 deficient Th2 cells compared with WT (Fig. 2C). Interestingly, significant reduction of HDAC1 recruitment was observed in NFAT1 deficient cells than WT upon stimulation (Fig. 5E). It seems that NFAT1 may act not only as an activator during the immediate early time (<1 h) after stimulation in WT cells, but also as an inhibitory molecule at later time points (>1 h) in Th2 cells (Fig.1), possibly through interaction of HDAC1. Few studies suggest the potential role of NFAT as a transcription repressor through interaction of HDAC molecules. A reduction in acetylation of histone H3 within the CDK4 promoter and direct association of NFAT1 with HDAC1 suppressed cyclin-dependent kinase 4 (*cdk4*) gene expression [37]. NFAT1 deficient mice showed increased expression of type II and type X collagen [38]. In addition, in our own studies on the transcription regulation of *IL-10* gene in T cells, we found that HDAC1 is constitutively associated with intron 4 [39] and promoter (Lee et al, unpublished) of the *IL-10* gene in Th1 cells, which may contribute to Th1-specific silencing of IL-10 expression in CD4^+^ T cells.

In the absence of NAFT1 which transcription factors mediate IL-4 expression by binding to the IL-4 promoter? To answer this question we performed EMSA-based Micro-LC/LC-MS/MS analysis and following proteins were identified such as special AT-rich sequence-binding protein-1 (SATB1), nuclear factor kappa-B (NF-κB), Jun-B oncogene (JUNB), NFAT, Jun oncogene (c-Jun), signal-transducer and activator of transcription protein (STAT) (Table 2). Interestingly, the list of identified proteins were matched well with the predicted data by bioinformatic analysis [40] (Fig. 4B). Among the identified proteins, JUNB, SATB1 and STAT were specifically enriched in NFAT1 deficient nuclear extract compared with WT Th2 cells (Fig. 4A). The appearance of this complex means that the NFAT1 complex is replaced at the IL-4 promoter by other transcriptional factor complexes after long term (6 h) stimulation. This complex might influence the regulation of IL-4 expression. JUNB also was identified as the partner of SATB1. JUNB is an abundant and inducible protein expressed in Th2 cells and has a cooperative role of SATB1 in IL-4 expression (Fig. 6A). Indeed, ChIP analysis showed the *in vivo* binding of JUNB and SATB1 at the IL-4 promoter (Fig. 5). Interestingly, preferential binding of JUNB was observed in NFAT1 deficient cells compared with WT Th2 cells while SATB1 binding levels were same between them (Fig. 5). In addition, no significant difference in the nuclear levels of SATB1 was observed between the cells (Fig. S3A). Among the JUNB and SATB1 proteins, JUNB may play a pivotal role in transactivation of the *IL-4* gene expression. Overexpression of JUNB significantly activated IL-4 promoter activity while knockdown of JUNB reduced IL-4 expression. In JUNB-mediated IL-4 promoter activation, SATB1 may play a synergistic role to activate JUNB function rather than transactivate IL-4 promoter activity by itself since overexpression of SATB1 alone failed to enhance IL-4 promoter activity (Fig. 6A). SATB1 organizes cell type specific nuclear architecture by anchoring specialized DNA sequences and recruiting chromatin remodeling factors to regulate gene transcription [41]. It is also required for expression of IL-4, IL-5, and IL-13 in a Th2 type specific manner by rapidly inducing a transcriptionally active chromatin structure at the cytokine locus [28], [29]. Therefore, recruitment of JUNB together with SATB1 may help to form an active chromatin complex at the IL-4 promoter. Indeed, enriched P300 and PCAF were observed at the IL-4 promoter in a stimulation dependent manner (Fig. 5). SATB1 interacts with both P300 and P300/CBP-associated factor (PCAF), a transcriptional coactivator with intrinsic histone acetylase activity [28]. Indeed, overexpression of transcription coactivators (P300 and PCAF) together with SATB1/JUNB further enhanced the IL-4 promoter activity (Fig. 6B). In addition, sustained IL-4 expression levels in NFAT1 deficient cells was well correlated with enhanced *in vivo* binding of PCAF, P300 and JUNB (Fig. 5) and acetylated histones (AcH3K9/14) (Fig. 2C) levels.

We also tested a possibility that in the absence of NFAT1, other NFAT members expressed in CD4 T cells could have compensatory function to mediate sustained IL-4 expression in NFAT1 deficient Th2 cells. However no significant increase of NFAT2 or NFAT4 was observed in NFAT1 deficient Th2 cells compared with WT (Fig. S5A). In line with the previously published data, NFAT1 deficiency did not alter the nuclear levels of JUNB and IRF4, the NF-κB family (p65 and p50) and Pol II (Fig. S5A). We also confirmed a similar nuclear JUNB level between WT and NFAT1 deficient Th2 cells by confocal microscopy analysis (Fig. S5B).

In conclusion, we have shown here that the sustained IL-4 expression in NFAT1 deficient Th2 cells is the result of permissive chromatin changes through hyperacetylation of histones and enhanced DNA demethylation in the IL-4 promoter. In addition, preferential binding of JUNB with transcriptional coactivators to the IL-4 promoter mediated sustained IL-4 expression in NFAT1 deficient Th2 cells.

## Materials and Methods

### Animals

C57BL/6 mice were purchased from SLC (Hamamatsu, Japan) and NFAT1^-/-^ mice were kindly provided by Dr. Anjana Rao (Boston, Harvard Medical School). Mice were housed in specific pathogen-free barrier facilities. All animal procedures were performed with the approval of Animal Care and Ethics Committees of the Gwangju Institute of Science and Technology (permit number: GIST-2008-12).

### Cell lines and primary T cells

Jurkat T cells E6.1 were purchased from American Type Culture Collection (ATCC, MA, USA) (Cat. Number; TIB-152). CD4^+^ T cells were purified from the spleen of 8-10-week-old female mice with the use of magnetic beads (L3T4 MicroBeads; Miltenyi Biotec, (Auburn, CA, USA)). For Th2 differentiation, naïve CD4^+^ cells (1×10^6^/ml) were stimulated with 1 µg/ml plate-bound anti-CD3ε under Th2-skewing (10 ng/ml IL-4, 10 µg/ml anti-IFN-γ plus 10 µg/ml anti-IL-12) conditions in Dulbecco's modified Eagle's medium (DMEM) supplemented with 10% fetal bovine serum, L-glutamine, penicillin-streptomycin, nonessential amino acids, sodium pyruvate, vitamins, HEPES, and 2-mercaptoethanol. At 24 h after stimulation, 10 U/ml recombinant human IL-2 (rhIL-2) was added, and the cells were expanded in complete medium containing IL-2 for 4 days. On day 6 after resting for 2 days, the cells were re-stimulated with plate-bound 1 µg/ml anti-CD3. Recombinant human IL-2 and IL-4 (11B11) were provided by the National Cancer Institute, Preclinical Repository. Anti-IFN-γ (XMG1.2) and anti-IL-12 (C17.8) were obtained from BD Biosciences (San Jose, CA) and anti-CD3 (145.2C11) and anti-CD28 (37.51) were from Pharmingen (San Diego, CA, USA).

### RNA isolation and quantitative real-time PCR

Total RNA was purified by using TRI Reagent purchased from Invitrogen. For reverse transcription, cDNA was generated by using 1 µg of total RNA plus oligo-dT and Improm-II Reverse Transcriptase (Promega, WI, USA) according to the manufacturer's instructions in a total volume of 20 µl. The mRNA level of IL-4 was determined by real-time PCR with SYBR green by using a protocol provided by the manufacturer (MJ Research Chromo 4). One microliter of cDNA was amplified by using the RT-PCR primer sets shown in Table 1. Expression levels were normalized to β-actin amplification levels in each sample.

### Chromatin accessibility by real-time PCR (CHART-PCR)

Chromaitn accessibility assays were performed as decribed [42] with minor modifications. Approximately 2×10^6^ nuclei in 100 ul nuclear digestion buffer (10 mM Tris-HCl pH 7.4, 15 mM NaCl, 60 mM KCl, 0.15 mM spermine, 0.5 mM spermidine, 1 mM CaCl_2_) plus 5 U/ml micrococcal nuclease (MNase; Roche, Basel, Switzerland) were incubated at 25°C for 10 min. Reactions were terminated with 20 µl stop solution (100 mM EDTA, 10 mM EGTA pH 8.1) and 10 µl 10% (w/v) SDS. DNA was isolated using a DNA blood genomic prep kit (Intron, Deageon, Korea) and eluted into 100 µl TE. DNAs recovered from MNase samples were checked for fragmentation in a 1% agarose gel. Untreated MNase samples were used in PCR assays to measure the relative abundance of target regions by using the primer sets shown in Table 1. To calculate the Ct value of each primer set, a standard curve was generated by using serial dilutions of genomic DNA. Chromatin accessiblity values were calculated as a ratio of the undigested sample to the digested samples, and then the data were plotted as the ratio of acessibility observed in the unstimulated digested DNA samples.

### Chromatin immunoprecipitation assay

The chromatin immunoprecipitation (ChIP) assay was performed essentially as described [39] with minor modifications. ChIP analysis was performed against SATB1, JUNB, P300, PCAF, and HDAC1 from Abcam (Cambridge, UK), RNA Pol II (Millipore, Temecula, CA; 05-623) and AcH3K9/27, H3K27me3, or rabbit IgG antibodies from Upstates (Temecula, CA, USA). Methylated DNA immunoprecipitation was performed as previously described [32]; [33] using anti-5-methylcytosine (anti-5mC) antibodies (Diagenode, Liege, Belgium). Fragmented DNA by Bioruptor was immunoprecipated using anti-5mC purchased from Diagenode (Liege, Belgium). Samples were used for RT-PCR analysis. Three independent experiments were performed. As a loading control, PCR was performed directly on input DNA purified from chromatin before immunoprecipitation. Quantification of IL-4 promoter fragment following ChIP was performed by real-time quantitative PCR. For normalization, 1% of ‘input’ DNA from each sample was analyzed in parallel and the amount of IL-4 DNA in each sample was calculated using the equation 2-^(Ct^_sample_^-Ct^_input_^)^^[43]^, where C_t_ is cycle threshold [43]. Real-time PCR detection system was carried out in triplicate. Primer sequences are shown in Table 1. ChIP assays were used to ensure that C_t_ values from samples DNA with specific antibodies resulted from specific immunoprecipitation.

### Pyrosequencing analysis

The promoter region of IL-4 (−450 ∼ −310) was amplified by using the forward primer and the biotinylated reverse primer (Table 1) designed by PSQ Assay Design (Biotage AB, Uppsala, Sweden). Genomic DNA (20 ng) was modified by sodium bisulfite with the EZ DNA Methylation kit (ZYMO Research, CA, USA) according to the manufacturer's instructions. Bisulfite-modified DNA was amplified in a 25 µl reaction with the primer set and 5 U/reaction of Taq polymerase (Solgent Co., Daejeon, Korea). Samples were heated to 95°C for 10 min and were then amplified for 40 cycles consisting of 95°C for 45 s, 55°C for 35 s, and 72°C for 60 s. All reactions were then incubated at 72°C for 10 min and cooled to 4°C. The PCR products were visualized on a 1.5% agarose gel by ethidium bromide staining for verification. Pyrosequencing reactions were done with sequencing primers on the PSQ HS 96A System (Biotage AB, Anaheim, CA) according to the manufacturer's specifications. The methylation index of each gene promoter and of each sample was calculated as the average value of ^m^ *C*~(^m^ *C*+*C*) for all examined CpGs in the target region. Statistical correlations between the methylation index and the clinical variables recorded were made by using SPSS version 11.

### Computational analysis of the conserved nucleotide sequence locus

Analysis of the sequences for transcription factor binding sites was performed with the MatInspector professional program (Genomatix Software, Munich, Germany) [40] by using the selected matrix library (vertebrate section) and optimized thresholds.

### Nuclear extract isolation and electrophoretic mobility shift assay

Electrophoretic mobility shift assay (EMSA) analysis was performed by using oligonucleotides corresponding to sequences in the NFAT/κb site in the IL-4 promoter. The oligonucleotides shown in Table 1 and the consensus κB oligonucleotide were purchased from Promega (Madison, WI). Complementary oligonucleotide pairs were annealed in 100 mM NaCl, 10 mM Tris pH 8.0, 0.1 mM EDTA buffer by heating to 95°C for 10 min and cooling slowly to the annealing temperature. Double-stranded oligonucleotides were end-labeled with [γ-^32^P] ATP and T4 polynucleotide kinase (Promega, Madison, WI). Labeled oligonucleotides were purified by use of a microspin G-50 column (275330, Amersham Biosciences, Uppsala, Sweden). Nuclear extracts were prepared from Jurkat cells stimulated for 2 h with phorbol myristate acetate (PMA)/ionomycin (P/I) and Th2 cells stimulated with anti-CD3 for 6 h. Twenty million cells were washed in PBS and suspended in buffer A (10 mM HEPES, 1.5 mM MgCl_2,_ 10 mM KCl, 0.5 mM DTT; 500 ml) with protease inhibitor cocktail (Roche: Mannheim,, Germany) and 5 mM β-glycerol phosphate. After 5 min of incubation on ice, release of nuclei was assessed by Trypan blue staining. Nuclei were sedimented in a microcentrifuge for 10 s at 4°C and were then resuspended in 2 pellet volumes of buffer C (20 mM HEPES, 25% glycerol, 1.5 mM MgCl_2_, 0.2 mM EDTA, 0.5 mM DTT, with inhibitor cocktail). The total volume of the nuclei was measured. One volume of buffer C containing 840 mM NaCl was added, and then nuclei were extracted for 20 min on ice, with occasional mixing. After determination of protein concentrations of the nuclear extracts by BCA assay (Bio-Rad: (Benicia-CA, USA)), nuclear extract (10 ug) was added to binding buffer (10 mM Tris pH 7.5, 0.5 mM MgCl_2_, 80 mM NaCl, 2.5 mM DTT, 4% glycerol, 1 mM β-mercaptoethanol, 20 ml) in the presence or absence of the labeled probes. For the competition assay, a 20-fold molar excess of the unlabeled probe was added and the samples were preincubated for 20 min. After 1 h of incubation on ice, the binding complexes were run in a 5% polyacrylamide gel in 0.25 TBE buffer (50 mM Tris, Boric acid, 1 mM EDTA pH 8, 4% glycerol) at 30 mA for 3 h at 4°C following pre-running at the same condition for 1 h.

### Transient transfection assay and plasmids

IL-4 minimal promoter locus (−632∼327) was amplified by PCR and cloned into pGL4 basic vector. SATB1/JunB and cofactors, p300 or/and PCAF expression plasmids were obtained from Korean Unigene Information (KUGI, Daejeon, Korea), and DNA sequences of each vector were confirmed by DNA sequencing analysis (Solgent Co., Daejeon, Korea). HEK cells were transfected by using Fugene 6 (Roche, Germany). An amount of 0.3 µg of the promoter luciferase reporter vector and 0.1 µg of pRL-TK resuspended in 100 µl containing 0.6 µl Fugene6 reagents was added to 2×10^6^ HEK-T cells. After a 16 h culture, rested cells were treated with 50 ng/ml PMA and 1 µM ionomycin for 6 h, and luciferase activity was measured by the dual luciferase assay system (Promega: Madison, WI) according to the manufacturer's instructions. Data were normalized by the activity of Renilla luciferase.

### Micro-LC/LC-MS/MS Analysis

To ideintify and charactrize the transcription factors bound to IL-4 promoter, micro-LC/LC-MS/MS analysis was performed essentially as described [44] with minor modifications. The nuclear extracts in Jurkat T cells or Th2 cells differentiated above mentioned method were prepared. After running, polyacrylamide gel were isolated corresponding to X-film band and then gel bands showing differential mobility shift were cut and analyzed by micro-LC/LC-MS/MS analysis. The identity of peptides were analyzed by protein database search [45].

### Statistical analysis

P values of <0.05 obtained with a two-tailed Student *t*-test were considered significant. Single asterisks (*) indicate p<0.05; double asterisks (**) indicate p<0.005.

## Supporting Information

Figure S1 **Sustained IL-4 expression in NFAT1 deficient Th2 cells.** (A) Th2 cells differentiated from WT or NFAT1 deficient CD4^+^T cells were left without stimulation or stimulated with anti-CD3 (α-CD3) for indicated time periods. The relative amount of nuclear NFAT1 protein levels was analyzed by Western blot. The effect of cyclosporine A (CsA), a calcineurin inhibitor, was also measured by adding CsA 30 min before stimulation. Lamin B level was measured as an internal control for nuclear protein extract. (B) The amount of IL-4 protein level from Th2 cells stimulated with α-CD3 for 36 h was measured by ELISA (B). Data shown are the mean ± SEM, from four separate experiments and * P<0.05.
(TIF)

Figure S2 **Increased DNA demethylation status at the IL-4 promoter of NFAT1 deficient Th2 cells.** Th2 cells from WT or NFAT1 KO mice were stimulated with anti-CD3 for 6 h or left without stimulation. DNA methylation state at the IL-4 promoter was analyzed by pyrosequencing. (A) The two targeted cytosines are underlined in original and converted sequences. (B) ‘T’ peaks (arrowed) indicate methylated cytosine while ‘C’ indicates unmethylated cytosine. The positive control, non-CpG cytosine residue showing complete conversion of cytosine to uracil by bisulphite treatment (asterisk) and non reactive C residue in yellow as negative control. First cytosine residue is unchanged in WT and NFAT KO upon stimulation, while second cytosine residue demonstrates a significant change in methylation. The Methylation Index (MtI) percentage is calculated as the average rate of G incorporation at each CpG. One representative of three independent experiments is shown.
(TIF)

Figure S3 **Constitutive expression of SATB1.** Th2 cells differentiated from WT or NFAT1 deficient CD4^+^ T cells were left without stimulation or stimulated with anti-CD3 for 6 h. The relative level of nuclear SATB1 was analyzed by Western blot. Tubulin and LaminB1 were used as controls.
(TIF)

Figure S4 **Pivotal role of JUNB in** ***IL-4*** **gene expression.** (A) Th2 cells from WT or NFAT1 deficient were left without stimulation or stimulated for 6 h. The expression levels of JunB and IL-4 were measured by agarose gel electophoresis. (B) Th2 cells from WT and NFAT1 deficient mice were transfected with scrambled mock siRNA (Mock) or JunB specific siRNA (si-JunB) and then specific knockdown efficiency of JunB and its effect on IL-4 expression level was measured by agarose gel electrophoresis.
(TIF)

Figure S5 **No significant increase of NFAT2, NFAT4, JUNB and other transcription factor in NFAT1 deficient Th2 cells.** (A) Th2 cells from WT or NFAT1 deficient were left without stimulation or stimulated for 6 h and then relative levels of diverse transcription factors using nuclear extract were compared by Western analysis. (B). Immunocytochemistry was performed to compare the nuclear levels of JUNB between WT and NFAT1 deficient Th2 cells after stimulation with α-CD3 for 0 or 6 h. FluoView microscope was used to analyze the stained cells; JUNB (FITC, blue), 7-Aminoactinomycin D (7-AAD, nuclear, red), merged (pink) and DIC (differential interference contrast).
(TIF)
