# Supplementary figures and images for: Enhanced Chromatin Accessibility and Recruitment of JUNB Mediate the Sustained IL-4 Expression in NFAT1 Deficient T Helper 2 Cells
Source: PLoS One. 2011 Jul 25;6(7):e22042. doi: 10.1371/journal.pone.0022042 (PMC3143129; doi:10.1371/journal.pone.0022042)

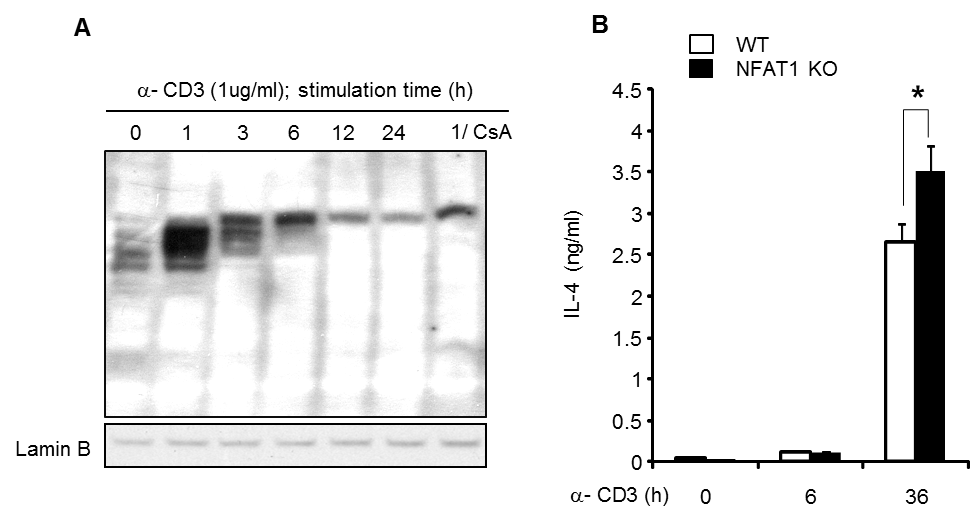

Supplement: Figure S1 — Sustained IL-4 expression in NFAT1 deficient Th2 cells. (A) Th2 cells differentiated from WT or NFAT1 deficient CD4+T cells were left without stimulation or stimulated with anti-CD3 (α-CD3) for indicated time periods. The relative amount of nuclear NFAT1 protein levels was analyzed by Western blot. The effect of cyclosporine A (CsA), a calcineurin inhibitor, was also measured by adding CsA 30 min before stimulation. Lamin B level was measured as an internal control for nuclear protein extract. (B) The amount of IL-4 protein level from Th2 cells stimulated with α-CD3 for 36 h was measured by ELISA (B). Data shown are the mean ± SEM, from four separate experiments and * P<0.05. (TIF) [file pone.0022042.s001.tif]

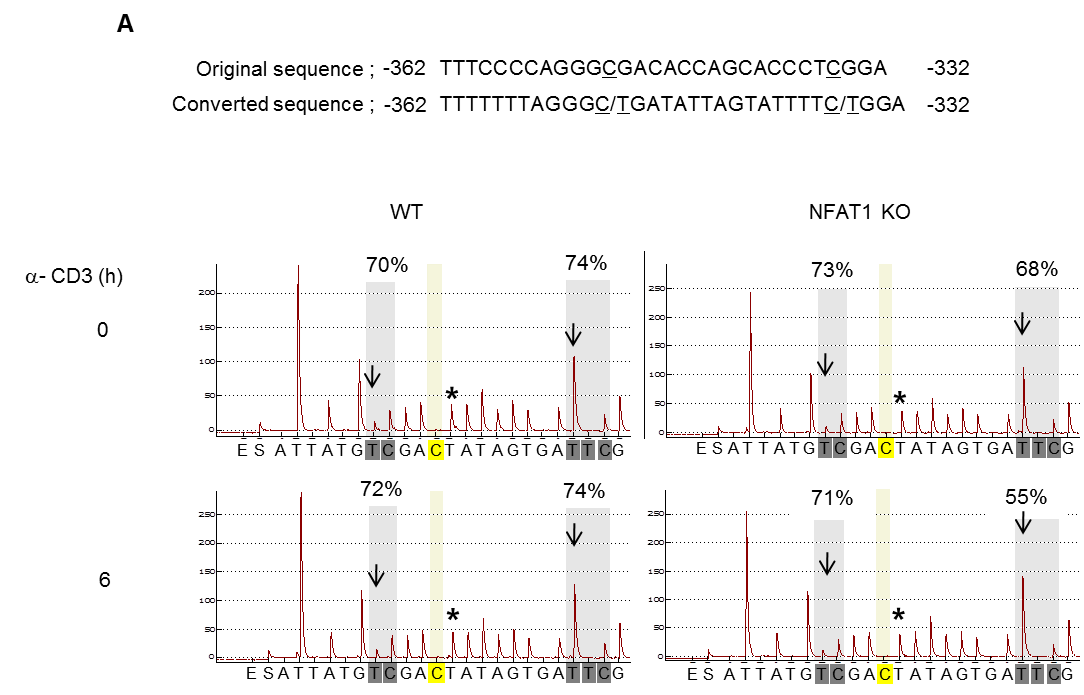

Supplement: Figure S2 — Increased DNA demethylation status at the IL-4 promoter of NFAT1 deficient Th2 cells. Th2 cells from WT or NFAT1 KO mice were stimulated with anti-CD3 for 6 h or left without stimulation. DNA methylation state at the IL-4 promoter was analyzed by pyrosequencing. (A) The two targeted cytosines are underlined in original and converted sequences. (B) ‘T’ peaks (arrowed) indicate methylated cytosine while ‘C’ indicates unmethylated cytosine. The positive control, non-CpG cytosine residue showing complete conversion of cytosine to uracil by bisulphite treatment (asterisk) and non reactive C residue in yellow as negative control. First cytosine residue is unchanged in WT and NFAT KO upon stimulation, while second cytosine residue demonstrates a significant change in methylation. The Methylation Index (MtI) percentage is calculated as the average rate of G incorporation at each CpG. One representative of three independent experiments is shown. (TIF) [file pone.0022042.s002.tif]

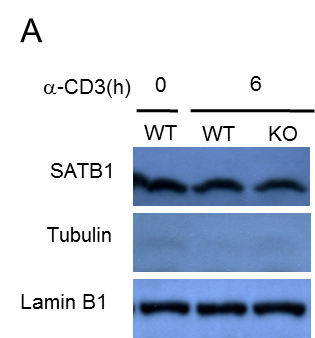

Supplement: Figure S3 — Constitutive expression of SATB1. Th2 cells differentiated from WT or NFAT1 deficient CD4+ T cells were left without stimulation or stimulated with anti-CD3 for 6 h. The relative level of nuclear SATB1 was analyzed by Western blot. Tubulin and LaminB1 were used as controls. (TIF) [file pone.0022042.s003.tif]

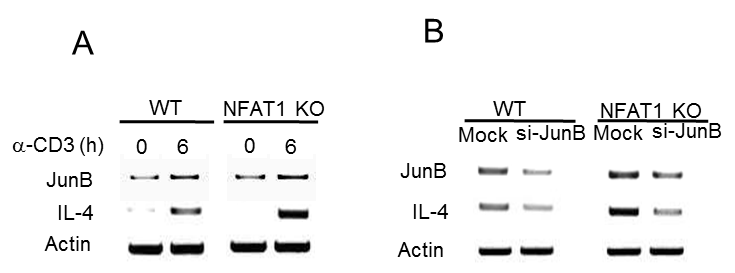

Supplement: Figure S4 — Pivotal role of JUNB in IL-4 gene expression. (A) Th2 cells from WT or NFAT1 deficient were left without stimulation or stimulated for 6 h. The expression levels of JunB and IL-4 were measured by agarose gel electophoresis. (B) Th2 cells from WT and NFAT1 deficient mice were transfected with scrambled mock siRNA (Mock) or JunB specific siRNA (si-JunB) and then specific knockdown efficiency of JunB and its effect on IL-4 expression level was measured by agarose gel electrophoresis. (TIF) [file pone.0022042.s004.tif]

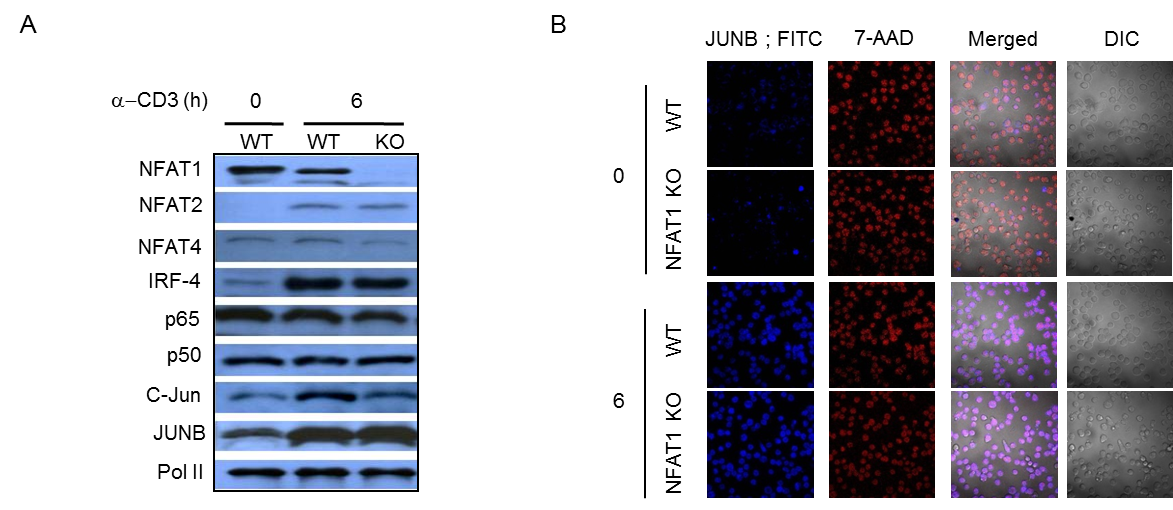

Supplement: Figure S5 — No significant increase of NFAT2, NFAT4, JUNB and other transcription factor in NFAT1 deficient Th2 cells. (A) Th2 cells from WT or NFAT1 deficient were left without stimulation or stimulated for 6 h and then relative levels of diverse transcription factors using nuclear extract were compared by Western analysis. (B). Immunocytochemistry was performed to compare the nuclear levels of JUNB between WT and NFAT1 deficient Th2 cells after stimulation with α-CD3 for 0 or 6 h. FluoView microscope was used to analyze the stained cells; JUNB (FITC, blue), 7-Aminoactinomycin D (7-AAD, nuclear, red), merged (pink) and DIC (differential interference contrast). (TIF) [file pone.0022042.s005.tif]
